# Transcriptome Analysis Reveals Key Gene Expression Changes in Blue Catfish Sperm in Response to Cryopreservation

**DOI:** 10.3390/ijms23147618

**Published:** 2022-07-10

**Authors:** Haolong Wang, Helen R. Montague, Hana N. Hess, Ying Zhang, Gavin L. Aguilar, Rex A. Dunham, Ian A. E. Butts, Xu Wang

**Affiliations:** 1 Department of Pathobiology, College of Veterinary Medicine, Auburn University, Auburn, AL 36849, USA; hzw0088@auburn.edu (H.W.); yzz0207@auburn.edu (Y.Z.); 2 Alabama Agricultural Experiment Station, Auburn, AL 36849, USA; hrm0028@auburn.edu (H.R.M.); hzh0074@auburn.edu (H.N.H.); gla0012@auburn.edu (G.L.A.); dunhara@auburn.edu (R.A.D.); 3 School of Fisheries, Aquaculture and Aquatic Sciences, Auburn University, Auburn, AL 36849, USA; 4 HudsonAlpha Institute for Biotechnology, Huntsville, AL 35806, USA

**Keywords:** *Ictalurus furcatus*, *Ictalurus punctatus*, hybrid catfish, sperm storage, sperm quality, RNA sequencing, motility, oxidative stress, DNA fragmentation, apoptosis

## Abstract

The hybrids of female channel catfish (*Ictalurus punctatus*) and male blue catfish (*I. furcatus*) account for >50% of US catfish production due to superior growth, feed conversion, and disease resistance compared to both parental species. However, these hybrids can rarely be naturally spawned. Sperm collection is a lethal procedure, and sperm samples are now cryopreserved for fertilization needs. Previous studies showed that variation in sperm quality causes variable embryo hatch rates, which is the limiting factor in hybrid catfish breeding. Biomarkers as indicators for sperm quality and reproductive success are currently lacking. To address this, we investigated expression changes caused by cryopreservation using transcriptome profiles of fresh and cryopreserved sperm. Sperm quality measurements revealed that cryopreservation significantly increased oxidative stress levels and DNA fragmentation, and reduced sperm kinematic parameters. The present RNA-seq study identified 849 upregulated genes after cryopreservation, including members of all five complexes in the mitochondrial electron transport chain, suggesting a boost in oxidative phosphorylation activities, which often lead to excessive production of reactive oxygen species (ROS) associated with cell death. Interestingly, functional enrichment analyses revealed compensatory changes in gene expression after cryopreservation to offset detrimental effects of ultra-cold storage: MnSOD was induced to control ROS production; chaperones and ubiquitin ligases were upregulated to correct misfolded proteins or direct them to degradation; negative regulators of apoptosis, amide biosynthesis, and cilium-related functions were also enriched. Our study provides insight into underlying molecular mechanisms of sperm cryoinjury and lays a foundation to further explore molecular biomarkers on cryo-survival and gamete quality.

## 1. Introduction

The human population is projected to reach 9.6 billion by 2050 [1], and the world demand for animal-derived protein might increase by 56% by 2050 [2,3]. To feed the global population, food animal production must increase by 60%. However, the supply of both grazing and cropland continues to decrease [4], and wild-caught fish stocks are also declining. Aquatic products, including fish, crustaceans, mollusks, and other aquatic animals, account for approximately 17% of total animal protein consumed by the global population [5], totaling 179 million tonnes, with a first-sale value of $401 billion USD [6]. Among these, aquaculture or fish farming represents nearly half (47–53%) of the total harvest [5]. As a fast-growing sector, aquaculture offers tremendous opportunities for biotechnological innovations to satisfy animal protein demands [7]. A total of 39 million kilograms of seafood and 248 million kilograms of freshwater species were harvested in 2016 in the US, ranking 16th worldwide. Catfish is the most important species in US aquaculture, and catfish farming in Mississippi, Alabama, Arkansas, and Texas accounts for 70% of total US freshwater aquaculture production.

The hybrid channel catfish, *Ictalurus* *punctatus*, ♀ × blue catfish, *I. furcatus*, ♂ primarily developed along with the embryo production technology at Auburn University [8,9,10,11,12,13], constitutes more than 50% of the total harvest in the US market [14]. This specific reciprocal is superior in pond culture in many aspects, including improved feed conversion efficiency [15,16], more carcass yield [17], better low oxygen tolerance [18], disease resistance [19], and enhanced harvestability [20]. Collectively, these heterobeltiosis characteristics enable a commercial production rate of 13,000 kg/ha, which doubles the yield of traditional channel catfish farming [16,21,22].

The bottleneck of hybrid catfish breeding is high-quality sperm production because sperm quality assessment and male gamete management are major challenges in catfish breeding. Blue catfish males reach sexual maturity at 4–7 years [23]. Unlike other fish in which sperm can be extracted by stripping, blue catfish sperm is collected through a lethal testes removal procedure in the industry [24]. The fact that milt can only be stripped once requires a substantial investment in sperm. In contrast, female channel catfish reach sexual maturity earlier (~3 years), and eggs can be readily hand-stripped for IVF [25]. These paternal complications make male gametes extremely precious in catfish production, and high-quality sperm can even be more valuable than eggs in reproduction [26]. Sperm preservation can facilitate the successful breeding of hybrid catfish. Sperm cryopreservation protocols have been developed for commercial-scale applications to provide year-round sperm when females are in peak spawning conditions [27,28,29]. In this regard, the USDA-ARS Animal Germplasm Program houses a large collection of frozen blue catfish sperm specimens in perpetuity. Unfortunately, a high degree of individual variability among fresh, cryopreserved, and refrigerated sperm was reported, resulting in huge variations in hatch rate (0 to 82%) in the farming practice [30,31,32,33,34]. This huge variation in sperm quality is hindering hybrid catfish embryo production.

Since high-quality gametes are the prerequisites for improved catfish reproduction, understanding the physiological and molecular mechanisms associated with sperm quantity/quality is critical for the accurate prediction of sperm fertility and offspring performance in hatchery environments. Sperm morphology, including size and shape, was shown to be associated with velocity and fertilization success [35]. Sperm concentration is also highly correlated with quality [36]. Oxidative stress causes apoptosis and mitochondrial function impairment during spermatogenesis, which directly impacts sperm quality [37]. In addition, seminal fluid is essential to sperm motility and function, and consequently, the sub-optimal composition of seminal fluid can lead to lower sperm quality [31]. While these findings by us and others collectively suggest physiological and cellular processes are linked to sperm quality, the underlying molecular mechanisms remain unclear.

In this study, we measured sperm kinematic parameters, DNA fragmentation, and oxidative stress levels before and after cryopreservation to determine the physiological changes in blue catfish sperm. RNA extraction and mRNA-seq experiments were performed for selected blue catfish sperm samples to investigate the alterations in global gene expression in response to cryopreservation. This study combined sperm quality parameters and transcriptomic profiles to contribute to a deeper understanding of the molecular mechanisms of cryoinjury and the response in fish sperm.

## 2. Results

### 2.1. Cryostorage Affects Sperm Motility, Increases the Level of Oxidative Stress, and Causes DNA Damage and Apoptosis 

**Figure 1:**
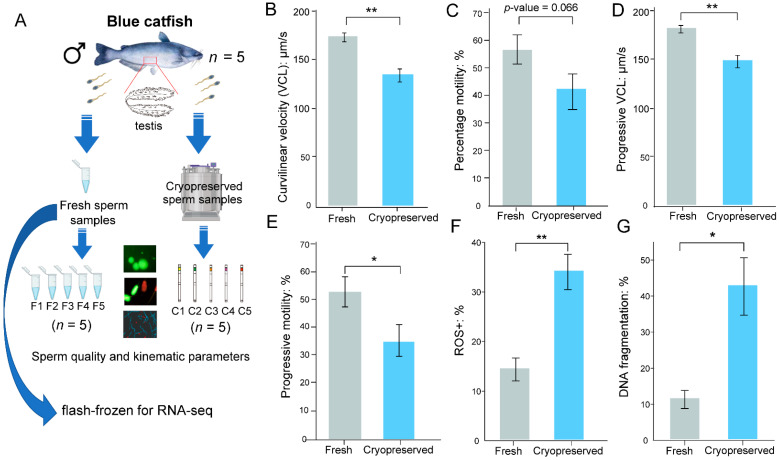
Blue catfish sperm quality metrics before and after cryopreservation. (**A**) Experimental design and procedure for comparing fresh (F) vs. cryopreserved (C) sperm quality. Various quality metrics were measured in fresh and cryopreserved sperm: curvilinear velocity (VCL) of motile sperm (**B**); percent motility (**C**), progressive VCL (**D**), progressive motility (**E**), oxidative stress level measured by percent of ROS+ (**F**), and DNA fragmentation (**G**). Statistical significance was determined by Student’s paired *t*-test (* *p* < 0.05; ** *p* < 0.01).

Common sperm characteristics between fresh and cryopreserved sperm samples were compared (Figure 1A). As shown in Figure 1, curvilinear velocity (VCL; Figure 1B), progressive VCL (Figure 1D), and progressive motility (Figure 1E) significantly decreased after cryopreservation (all *p*-values < 0.05, paired *t*-test). Percent motility was marginally significant at *p* = 0.066 (Figure 1C). The oxidative stress level of fresh sperm was approximately 3-fold lower than cryopreserved sperm, with a *p*-value of 0.002 (Figure 1F). The percentage of DNA fragmentation in fresh sperm was approximately 4-fold lower than that in cryopreserved sperm (*p*-value = 0.014; Figure 1G). Taken together, our findings indicated that fresh sperm quality decreased after cryopreservation.

### 2.2. RNA Sequencing Revealed 849 Upregulated and 143 Downregulated Genes in the Blue Catfish Sperm Transcriptome after Cryopreservation 

**Figure 2:**
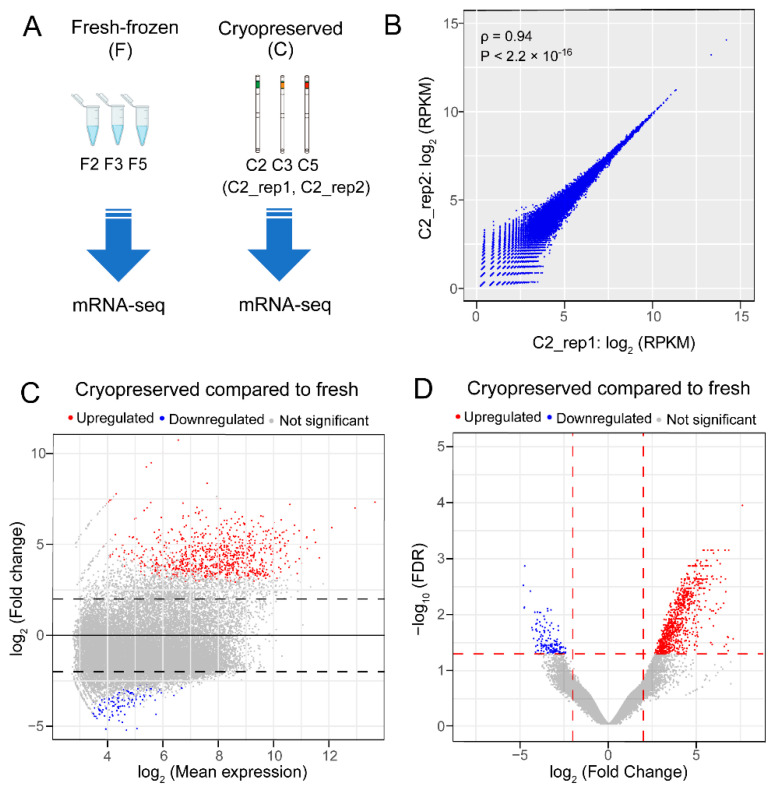
Transcriptome-wide identification of differentially expressed genes (DEGs) between the fresh-frozen and cryopreserved sperm in the blue catfish, *Ictalurus furcatus*. (**A**) RNA-seq samples were fresh-frozen (F) and cryopreserved (C) sperm samples from three males with ID numbers 2, 3, and 5. C2_rep1 and C2_rep2 are two technical replicates of the C2 sample. (**B**) Scatterplot of the log_2_(RKPM) values for C2_rep1 and C2_rep2. Spearman’s rank correlation coefficient ρ and the corresponding *p*-values are labeled. (**C**) MA plot of the comparison between fresh-frozen and cryopreserved sperm. Horizontal dash lines indicate |log_2_(fold change)| = 2, and DEGs are highlighted in red (upregulated) and blue (downregulated) dots. (**D**) Volcano plot of the comparison between fresh-frozen and cryopreserved sperm. The vertical dash lines indicate |log2(fold change)| = 2 and the horizontal line represents False Discovery Rate (FDR) < 0.05. Upregulated DEGs and downregulated DEGs were highlighted in red and blue, respectively.

As shown in Figure 2A, transcriptome analyses were conducted on three biological replicates of fresh-frozen sperm samples (F2, F3, and F5) and three biological replicates of cryopreserved sperm samples (C2, C3, and C5). On average, ~80% of the RNA-seq reads were aligned to the blue catfish reference genome (Table S2). A total of 27,416 expressed transcripts were detected with an average Reads Per Kilobase of transcript per Million mapped reads (RPKM) value greater than 1.0 in at least one treatment group. To validate the reproducibility of RNA-seq for the same sample, two technical replicates of C2 samples were included (C2_rep1 and C2_rep2). The gene expression values of these two replicates were significantly correlated with a Spearman’s rank correlation coefficient ρ = 0.94 (*p*-value < 2.2 × 10^−16^), indicating high reproducibility in total RNA extraction, library preparation, and RNA sequencing (Figure 2B). To identify differentially expressed genes (DEGs), a pairwise differential gene expression analysis was performed between the fresh-frozen and cryopreserved groups. The number of upregulated DEGs and downregulated DEGs were 849 and 143, respectively (Figure 2C,D and Figure S1, Data S1). Notably, the upregulated DEGs have significantly greater fold change than the downregulated genes (Figure 2C,D and Figure S1). Collectively, the transcriptome pattern suggested a global upregulation of gene expression in response to cryopreservation. 

### 2.3. Quantitative Reverse Transcription Polymerase Chain Reaction (qRT-PCR) Validation of Differentially Expressed Genes 

**Figure 3:**
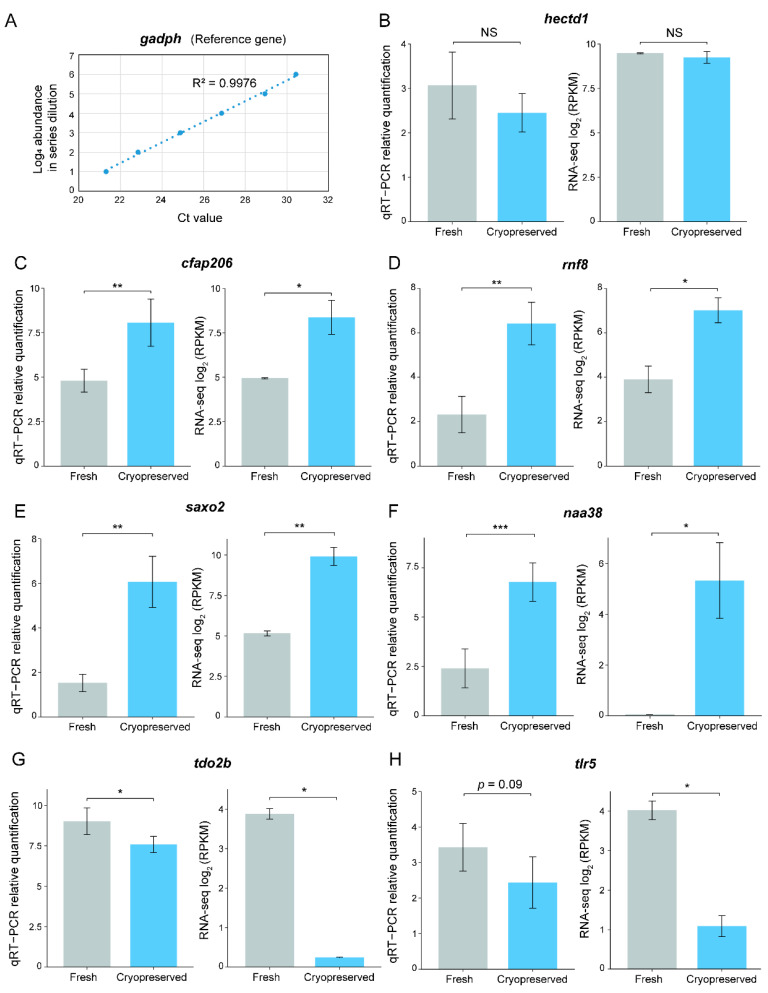
Quantitative reverse transcription PCR validation of differentially expressed genes (DEGs) in fresh-frozen and cryopreserved sperm in the blue catfish, *Ictalurus furcatus*. (**A**) Scatterplot of the standard curve based on a series of dilutions for the reference gene *gapdh*. (**B**–**H**) Barplots of qRT-PCR relative quantification (***left***) and log_2_ RNA-seq RPKM values (***right***) for *hectd1* (**B**), *cfap206* (**C**), *rnf8* (**D**), *saxo2* (**E**), *naa38* (**F**), *tdo2b* (**G**), and *tlr5* (**H**). Student’s *t*-test was used to assess the statistical significance (* *p* < 0.05; ** *p* < 0.01; *** *p* < 0.001).

The relative expression levels of seven genes were validated by qRT-PCR with a housekeeping gene *gadph* as reference (Figure 3A and Table S3), including a non-differentially expressed gene *hectd1* (Figure 3B), four upregulated genes (*cfap206*, *rnf8*, *saxo2*, and *naa38*; Figure 3C–F), and two downregulated genes (*tlr5* and *tdo2b*; Figure 3G,H). Among them, RNA-seq expression levels of *cfap206*, *rnf8*, *saxo2*, and *naa38* were significantly increased after cryopreservation (FDR < 0.05), and they were all confirmed in the qRT-PCR experiments in the same direction (*p*-value < 0.05; Figure 3C–F). The downregulation of *tdo2b* was also verified (*p*-value < 0.05; Figure 3G). *trl5* was also downregulated in the RNA-seq data, and it was marginally significant in the qRT-PCR validation results (*p*-value = 0.09; Figure 3H). The result would achieve statistical significance (*p*-value < 0.05, one-sided *t*-test) if a one-sided test were performed, given the prior knowledge of downregulation in RNA-seq. 

### 2.4. GO Analyses Revealed Upregulated Functional Clusters after Cryopreservation

**Figure 4:**
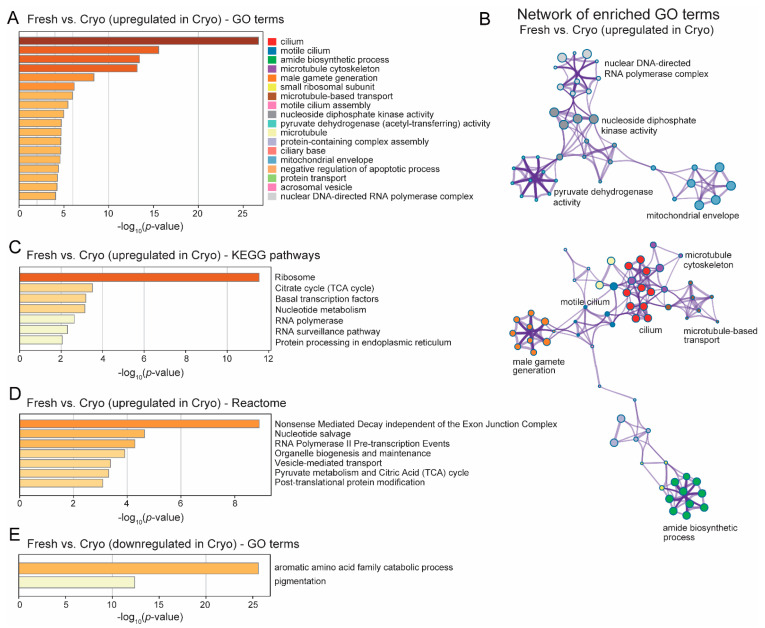
GO terms and pathway enrichment analyses of differentially expressed genes (DEGs) in cryopreserved sperm in blue catfish, *Ictalurus furcatus*. (**A**) Enriched functional GO terms for upregulated genes in cryopreserved sperm compared to fresh-frozen sperm with a cut-off of −log_10_(*p*-value) > 5. Enrichment scores measured by −log_10_(*p*-value) were shown on the *x*-axis. (**B**) Network plot of enriched GO terms of upregulated genes after cryopreservation. GO term nodes were represented by the same color as in (**A**). (**C**) Enriched functional KEGG pathways for upregulated genes in cryopreserved sperm compared to fresh-frozen sperm with a cut-off of −log_10_(*p*-value) > 2. (**D**) Enriched functional reactome pathways for upregulated genes in cryopreserved sperm compared to fresh-frozen sperm with a cut-off of −log_10_(*p*-value) > 3. (**E**) Enriched functional GO terms for downregulated genes in cryopreserved sperm compared to fresh-frozen sperm.

Gene Ontology (GO) enrichment analyses were performed to identify the altered biological functions and pathways of those DEGs between the two groups. A total of 18 GO terms were significantly enriched for upregulated genes in cryopreserved sperm (Figure 4A). Among them, 14 functional terms were tightly connected in two distinct gene networks (Figure 4B). The first upregulated cluster included ten terms: cilium, motile cilium, motile cilium assembly, ciliary base, microtubule, microtubule cytoskeleton, microtubule-based transport, acrosomal vesicle, amide biosynthetic process, and male gamete generation (Figure 4A,B), which were cellular structure and organelles responsible for sperm motility and transport. The second cluster involved four functional categories, nucleoside diphosphate kinase activity, and nuclear DNA-directed RNA polymerase complex, which were directly related to gene transcription, as well as mitochondrial envelope and pyruvate dehydrogenase activity (a mitochondrial multienzyme complex), which were mitochondrial functions (Figure 4B). The top four most enriched terms among upregulated genes were extremely significant, with a *p*-value < 10^−10^. Of these, three are sperm motility-related functions (cilium, motile cilium, and microtubule cytoskeleton), and the remaining one is the amide biosynthesis pathway (Figure 4B). Amides are organic chemicals with a carbonyl group attached to a nitrogen atom and are often used as a cryoprotectant in sperm preservation [38,39,40,41]. There was a fewer number of downregulated DEGs, and they were primarily enriched for the aromatic amino acid family catabolic process (Figure 4E).

### 2.5. The KEGG Pathway Enriched for Upregulated DEGs after Cryopreservation—mRNA Expression, Oxidative Phosphoralation and Mitochondrial ROS Regulation 

To explore molecular function and metabolic pathway activity alterations after cryo-preservation, Kyoto Encyclopedia of Genes and Genomes (KEGG) reference pathways were examined with respect to upregulated DEGs after cryopreservation. KEGG and reactome analyses also revealed functional terms related to RNA transcription and protein synthesis among upregulated genes (*p*-value < 0.01), including ribosome, nucleotide metabolism, RNA polymerase, protein processing in the endoplasmic reticulum (Figure 4C), as well as RNA polymerase II pre-transcription events, post-translational protein modification, metabolism of amino acids and derivatives (Figure 4D). Transcription regulation functions were also highly significant, including basal transcription factors, RNA surveillance, nucleotide salvage, and nonsense-mediated decay.

**Figure 5:**
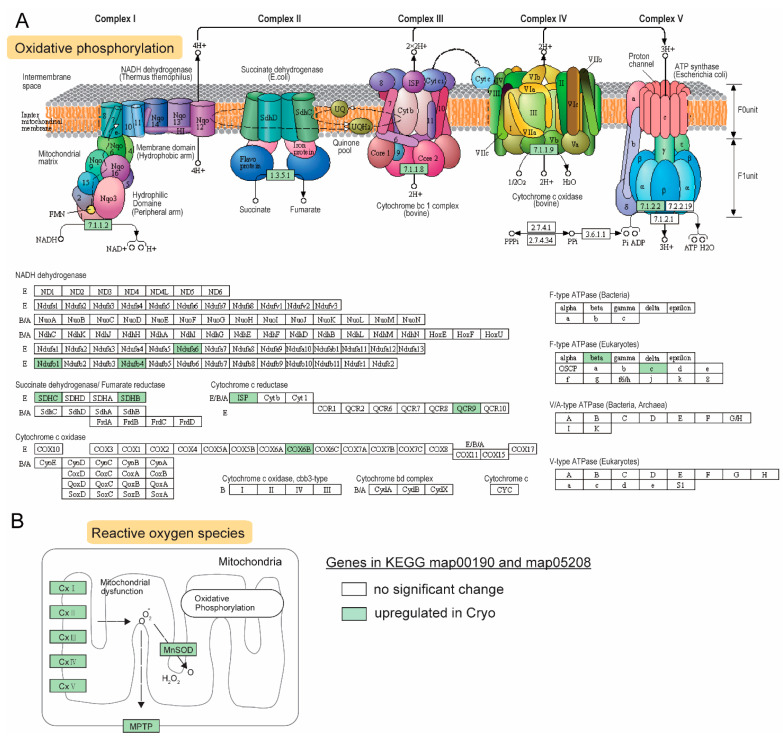
Effects of cryopreservation of sperm on mitochondrial oxidative pathways in blue catfish, *Ictalurus furcatus*. (**A**) KEGG map of the oxidative phosphorylation pathway in mitochondrial (KEGG ID: map00190). (**B**) KEGG map of chemical carcinogen and reactive oxygen species (KEGG ID: map05208). Upregulated genes, proteins, and enzymes were highlighted in green.

Upregulated DEGs were mapped to major reference pathways, including oxidative phosphorylation and reactive oxygen species. Notably, all five enzymatic complexes embedded in the inner membrane in the oxidative phosphorylation (OXPHOS) chain had DEGs (Figure 5A), suggesting an enhanced OXPHOS activity after cryopreservation. Pyruvate metabolism and citric acid cycle (TCA) genes were also enriched, which were associated with mitochondrial metabolism (Figure 4C,D). 

A higher level of oxygen metabolism can lead to excessive accumulation of by-products known as reactive oxygen species (ROS) [42], which could trigger and mediate downstream cellular processes such as apoptosis and adhesion, and alter gene expression [43]. Manganese superoxide dismutase (MnSOD) is a sensor and master regulator of ROS, and overexpression of MnSOD can increase the antioxidant capacity to combat the abnormal accumulation of ROS [44]. In our results, MnSOD was significantly upregulated after cryopreservation (log_2_ fold change = 3.07 and FDR < 0.05; Figure 5B). Another upregulated gene in the mitochondria was the mitochondrial permeability transition pore protein (mPTP), which is a protein on the inner membrane (Figure 5B). mPTP can be induced by Ca^2+^ and triggered by endoplasmic reticulum (ER) stress, leading to cell death via apoptosis or necrosis [45].

### 2.6. The KEGG Pathway Enriched for Upregulated DEGs after Cryopreservation—Heat Shock Proteins and Protein Ubiquitination/Degradation

**Figure 6:**
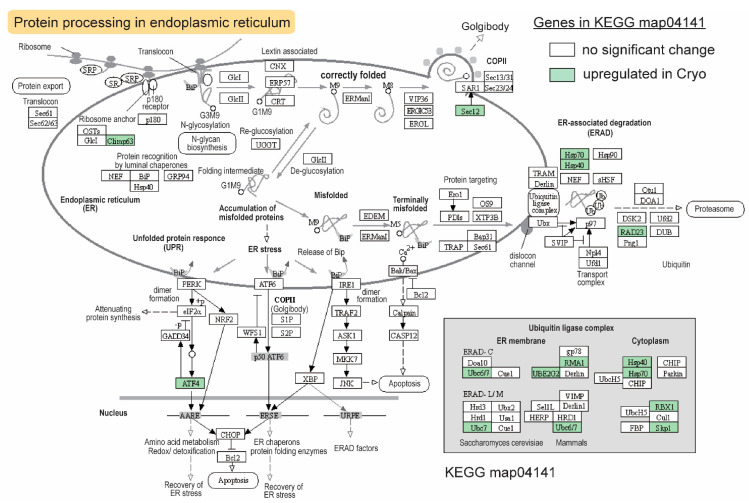
Effects of cryopreservation of sperm on protein processing in the endoplasmic reticulum (map04141) in the blue catfish, *Ictalurus furcatus*. Upregulated genes, proteins, and enzymes were highlighted in green.

Upregulated gene sets were found in the ER-associated protein degradation process (Figure 6). As important heat shock proteins (HSPs) and molecular chaperones, Hsp40 works with Hsp70 to perform critical functions in protein folding and degradation [46]. Both genes were significantly upregulated after cryopreservation (Figure 6). In addition to *hsp70* and *hsp40*, six members of the ubiquitin ligase complex genes were also upregulated, which encode Ube2g2 (Ubiquitin-Conjugating Enzyme E2 G2), Rma1 (E3 ubiquitin-protein ligase), Rad23 ubiquitin receptor, Ubc7 (Ubiquitin-Conjugating enzyme 7), Rbx1 (RING box protein 1), and Skp1 (S-phase kinase-associated protein 1), which are essential components of Skp1/Cullins/F-box E3 ubiquitin ligases (Figure 6). Other upregulated genes in this pathway included guanine nucleotide-exchange factor *sec12* involved in ER transport vesicle formation and autophagy, cyclic AMP-dependent transcription factor *atf4*, and *climp63* (cytoskeleton-associated protein 4; Figure 6). 

### 2.7. The KEGG Pathway Enriched for Upregulated DEGs after Cryopreservation—Apoptosis and Necroptosis Pathways

**Figure 7:**
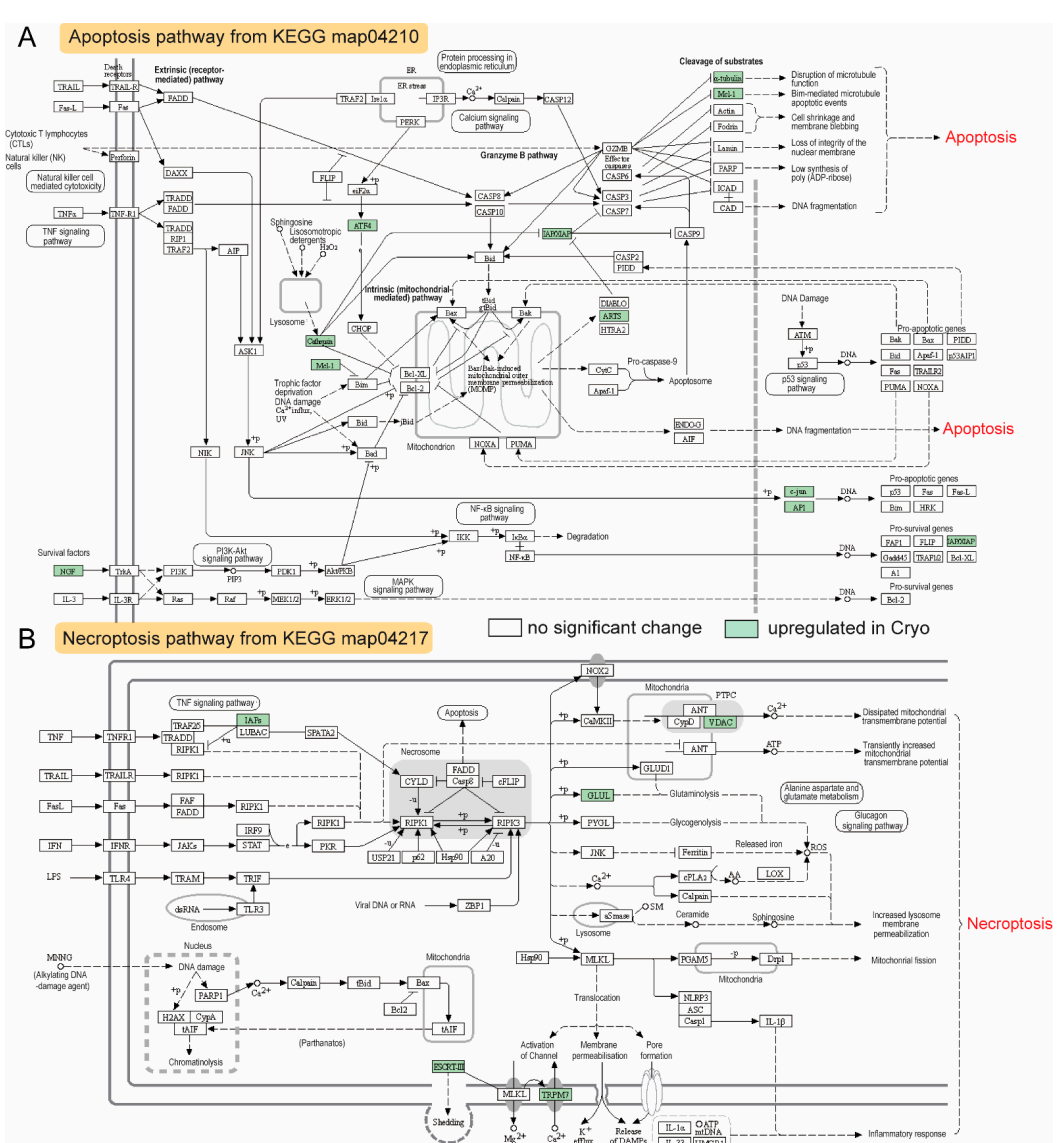
Effects of cryopreservation of sperm on the apoptosis and necrosis pathways in blue catfish, *Ictalurus furcatus*. (**A**) KEGG map of the apoptosis pathway (map04210). (**B**) KEGG map of necrosis (map04217). Upregulated genes, proteins, and enzymes were highlighted in green.

Cryopreservation also induced apoptosis-related genes and enzymes, including *mcl1*, *xiap*, *arts*, *ngf*, and *cathepsin* (Figure 7A). Another set of upregulated genes, *iaps*, *vdac*, *glul*, *escrt-iii*, and *trpm7*, play important roles in the necroptosis pathways, which may alter plasma membrane integrity and mitochondrial activity (Figure 7B).

## 3. Discussion

### 3.1. Sperm Quality Measurement Revealed Potential Impairment of Cryopreservation in Motility, DNA Fragmentation, and Level of Oxidative Stress 

Cryoinjury has been widely studied from a physiological and biochemical perspective. Cryopreservation has been shown to have deleterious impacts on the plasma membrane, mitochondria, chromatin structure, osmotic control, and spermatozoa motility [47,48]. In this study, the kinematic properties of blue catfish sperm after cryopreservation displayed reduced motility (Figure 1), which may reduce their fertilization potential. A 3-fold higher oxidative stress level was observed (Figure 1F), suggesting cryopreservation did impose a certain amount of stress, which may lead to apoptosis and mitochondrial function impairment [37]. Sperm DNA fragmentation level was 4-fold higher after cryopreservation (Figure 1G). These quality parameters indicated that the sperm quality might be impaired after cryopreservation. However, a more comprehensive assessment is needed using a much larger sample size. 

### 3.2. Cryopreservation on Sperm Transcriptome—Global Upregulation of Gene Expression, Increased Oxidative Phosphorylation, and Glycolysis/TCA Activities in the Mitochondria

The findings in the transcriptome analyses in fresh-frozen vs. cryopreserved blue catfish sperm revealed that ~900 genes were upregulated in response to cryopreservation. Genes involved in energy metabolism and adenosine triphosphate (ATP) production are enriched in our study after cryopreservation. Spermatozoa are highly specialized cells that require sufficient ATP to maintain normal physiological and biochemical processes, such as the synthesis of proteins, sperm motility, capacitation, and fertilization [49]. It was reported that sperm motility is dramatically reduced during cryopreservation [50]. In rainbow trout, the ATPase was significantly lower in frozen-thawed sperm [51]. The most crucial ATP sources are glycolysis, TCA, and oxidative phosphorylation pathways [52], which were shown to be enriched in upregulated genes (Figure 4). Consistent with our findings, the pyruvate metabolism pathway was also enriched in stallion sperm after cryopreservation [53]. In bovine sperm, it was reported that adding pyruvate to the sperm can reduce sperm mortality of cryopreserved sperm, although the ROS level was increased marginally [54]. In our results, increased gene expression levels were also discovered in all five OXPHOS enzyme complexes, indicating a higher level of mitochondria activity, which may lead to the overproduction of ROS observed in our sperm quality assessment results. Elevated oxidative stress level is often associated with cell death, and we did observe enriched terms of apoptosis and necroptosis. 

### 3.3. Compensatory Changes after Cryopreservation in Sperm Transcriptome to Improve Cilia Structure and Sperm Motility

Our physiological experiments revealed the potential impacts of cryopreservation. If all effects were detrimental, how come the cryopreserved sperm can still fertilize catfish eggs? This could be reconciled by in-depth investigations of the transcriptome changes. Cilium, motile cilium, motile cilium assembly, and other related GO terms were the most significantly enriched functional categories after cryopreservation (Figure 4A), which may partly restore sperm motility after cryopreservation. 

A significantly regulated gene validated by qRT-PCR, *saxo2* (Figure 3E), is a stabilizer of the axonemal microtubules family, which is found in ciliated or flagellated eukaryotes and may play a key role in cell motility. A gene in the same family, *saxo1*, is localized in mature human sperm and involved in microtubule binding. Knockdown of *saxo1* suggested that it modulates axoneme length in humans [55]. There was also evidence that *saxo1* expression was significantly decreased in asthenozoospermia compared to normal sperm [56]. The role of *saxo2* in sperm axoneme and sperm motility remains to be studied in fish. 

Another upregulated gene, *cfap206*, belongs to the cilia/flagella-associated protein family, which is reported to play an important role in the normal assembly of cilia or flagella [57]. Mutations in *cfap65* resulted in abnormal sperm flagellum in humans and mice, leading to male infertility [58]. Recently, the function of *cfap206* was studied in an aquaculture species (*Mulinia lateralis*) through RNAi. The expression of cfap206 was significantly decreased in the male gonad, and aberrant sperm tail assembly was observed, resulting in decreased sperm motility [59]. *cfap206* was also validated by qRT-PCR in our study (Figure 3C).

### 3.4. Compensatory Changes after Cryopreservation in Sperm Transcriptome—Negative Regulation of Apoptosis

Apoptosis is critical for normal spermatogenesis in eukaryotes to eliminate superfluous cells and maintain cellular homeostasis [60]. Cryopreservation was shown to induce apoptosis in humans [61], mice [62], and bovine spermatozoa [63]. In fish, apoptotic cells were significantly increased in Senegalese sole sperm after cryopreservation [64]. Negative regulation of the apoptotic process (*p* < 10^−5^; Figure 4A) was significantly enriched in our study, which could be the way to combat the apoptosis induced by cryopreservation. Among the identified DEGs, *bcl2l10* and *naa38* are the most important negative regulators of apoptosis. *Bcl2l10* is an anti-apoptotic member of the BCL-2 family, which is highly concentrated in mitochondria [65]. Bcl-2 can inhibit cytochrome C release and thus caspase activation, indicating that it protects the mitochondrial outer membrane’s integrity [66]. There is evidence that pro-apoptotic and anti-apoptotic family members can heterodimerize, and their relative concentrations may act as a switch for programmed cell death [67]. Naa38 is an auxiliary subunit of the NatC N-acetyltransferase complex. It is ubiquitously expressed in testis and is involved in the negative regulation of the apoptotic process. In humans, NAA38 was reported to interact with NAA30 and NAA35 in the NatC complex, and this complex was cosedimented with ribosomal pellets [68]. Our study showed the most enriched KEGG pathway for upregulated DEGs is ribosome (Figure 4C), suggesting that it might be involved in cotranslational acetylation. Knockdown of NatC subunits can lead to p53-dependent apoptosis [68]. 

### 3.5. Compensatory Changes after Cryopreservation in Sperm Transcriptome—Amide Biosynthesis

Amide is shown as an excellent cryoprotector for semen preservation in many organisms, including stallions [40,69], cats [39], boar [41], and fish [38]. Interestingly, the amide biosynthesis pathway is extremely enriched in upregulated genes (*p* < 10^−14^; Figure 4A) after cryopreservation. The amide biosynthesis pathway was also enriched in sheep testicular somatic cells during spermatogenesis, suggesting the potential roles in male gamete function [70]. 

### 3.6. Compensatory Changes after Cryopreservation in Sperm Transcriptome—Managing ROS by Upregulation of MnSOD

Elevated OXPHOS activities in the mitochondria may lead to excessive production of ROS [42], which triggers cell death [43]. The superoxide dismutases (SODs) family was the most critical line of defense system against ROS. Among these SODs, *sod2* encodes MnSOD enzyme, which plays a key role in immune function [71] and oxidative stress response [72]. In mice, *Sod2* knockout led to severe oxidative damage in the mitochondria [73]. In humans, it was reported that SOD activity was significantly reduced in the semen of male infertility compared to normal male semen [74]. Interestingly, It has been proven that SOD2 was increased in bull sperm after cryopreservation [75]. The *sod2* was significantly upregulated in cryopreserved sperm in our study, suggesting that the cryopreservation may induce antioxidant protection against oxidative stress and cell damage in blue catfish sperm.

### 3.7. Compensatory Changes after Cryopreservation in Sperm Transcriptome—ER-Stress-Related HSPs and Ubiquitin-Dependent Protein Degradation

Increased transcription and ribosome activities in response to cryopreservation (Figure 4) may lead to elevated pressure for correctly folding the newly synthesized proteins, which induces ER stress. If the misfolded proteins were not handled properly in a timely manner, apoptosis or necroptosis could occur. Heat shock proteins, including Hsp70, can provide cryoprotection to cells [76]. Two heat shock proteins (Hsp70 and Hsp40), together with a large number of members in the ubiquitin ligase complex, were upregulated after cryopreservation. They will resolve the ER-stress issue by folding the protein properly or guiding them for degradation in the proteasome. 

Rnf8, a RING domain protein, encodes an E3 ubiquitin ligase enzyme. It has been proven to play important roles in many biological processes, including DNA damage response and repair [77]. In mice, knockout of *rnf8* led to a failure in spermiation [78], suggesting that Rnf8 is required for spermatogenesis and that its absence could result in infertility. Intriguingly, the level of DNA fragmentation (Figure 1D) and the expression of *rnf8* (Figure 3D) were both increased after cryopreservation in our study, suggesting that *rnf8* may be used as a potential biomarker for evaluating sperm quality.

### 3.8. Compensatory Changes after Cryopreservation in Sperm Transcriptome—Potential Alterations in Immune Functions?

Inflammation has negative impacts on sperm quality [79]. The male reproductive system adopts a distinct immune activity to avoid harmful autoimmune responses. Testes, for example, have immune-privileged features that protect sperm from immunological attack during maturation and storage [80]. Therefore, upregulation of immune function is often associated with reduced sperm quality. In our qRT-PCR validation experiments, we confirmed the downregulation of *tlr5* after cryopreservation (Figure 3H), which is a Toll-like receptor. A TLR5 agonist was shown to protect against radiation-induced male reproductive system injury in mice [81]. Further investigations are required to elucidate the role of *tlr5* in blue catfish sperm quality. 

### 3.9. Aromatic Amino Acid Catabolic Process-Related Genes Were Downregulated in Blue Catfish Sperm after Cryopreservation

The only highly significant GO term for downregulated genes is aromatic amino acid metabolism (Figure 4E), including *hpda* (4-hydroxyphenylpyruvate dioxygenase a), *ocrl* (inositol polyphosphate 5-phosphatase), *tdo2b* (tryptophan 2,3-dioxygenase b), and *ido1* (indoleamine 2,3-dioxygenase 1). *hpda* is a key enzyme in tyrosine metabolism, and it was identified as a candidate gene related to pigmentation crucian carp [82], which is why pigmentation was identified as a significant GO term in our study (Figure 4E). *ocr1* belongs to the transient receptor potential vanilloid (TRPV) subfamily, which includes several heat sensors. In *C. elegans*, three TRPV channel subunits (*osm9*, *ocr1*, and *ocr2*) are involved in the acclimatization related to cold tolerance [83]. There was also evidence that sperm can affect the activity of temperature-sensing neurons through feedback network, which controls cold tolerance in *C. elegans* [84]. Loss of *ocrl* in Drosophila activates multiple immune signaling pathways, including Toll, Jun kinase, and STATs, which promotes the innate immunity function, potentially through endosomal signaling [85]. *tdo2b* is one of the key enzymes involved in tryptophan (Trp) metabolism [86], and its downregulation was confirmed in our qRT-PCR experiments (Figure 3H). *ido1*, a sister enzyme of Tdo, is the rate-limiting enzyme in tryptophan metabolism into downstream kynurenines, which has been widely studied in the context of immune regulation [87]. It was reported that *Ido*^−/−^ mice had an increased number of abnormal spermatozoa [88]. The mechanism of Trp metabolism’s influence on cryopreservation remains to be further explored, but it may relate to innate immune function [89]. 

### 3.10. Toward a Better Understanding of in Impact of Cryopreservation on Sperm, and Identification of Potential Biomarkers of Sperm Quality 

Our results revealed that, in blue catfish, sperm cryopreservation could affect a variety of biological processes, including but not limited to apoptosis, spermatogenesis, changes in cilia structure, mitochondrial activity, ROS production, amide biosynthesis, protein folding and degradation. The significantly upregulated genes involved in mitochondrial oxidative stress and protein degradation in the endoplasmic reticulum are interconnected (Figure 5 and Figure 6). Thus, the influence of cryopreservation is quite complex, with both detrimental and compensatory effects on sperm quality. In addition to gene expression level, cryopreservation can also affect DNA methylation. For example, 1266 differentially methylated genes (DMGs) were identified in black rockfish sperm methylome after cryopreservation, including 1005 upregulated DMGs [90]. Furthermore, non-coding RNAs (ncRNAs) and microRNAs (miRNAs) can regulate genes involved in spermatogenesis, affecting sperm quality [91,92]. Further studies using comprehensive sperm health metrics and offspring performance indices combined with multiomics approaches will help better understand the influence of sperm cryopreservation on sperm quality, and inform the discovery of robust biomarkers as sperm quality indicators for hybrid catfish breeding applications. 

## 4. Materials and Methods

### 4.1. Fish Maintenance, Sperm Sample Collection, and Cryopreservation

All experimental animal protocols were approved by the Auburn University Institutional Animal Care and Use Committee (AU-IACUC) with protocol number 2020–3710. Mature blue catfish males (body weight: 5.19 ± 1.01 kg and body length: 74.63 ± 7.72 cm) were provided by Jubilee Farms (Indianola, MS, USA), and transported to the E.W. Shell Fisheries Center (Auburn, AL, USA). These fish were cultured in 0.04-ha earthen ponds with a floating pond surface aerator. Water temperature ranged from 19.9 to 25.4 °C, and pH varied between 7.5 and 8.6. Dissolved oxygen was >6 mg/L, salinity was <2 ppm, and alkalinity and hardness ranged from 50 to 80 mg/L.

Five males were randomly selected and euthanized following industry-approved protocols. Testes were dissected from each male and then washed with Hank’s balanced salt solution (HBSS) [93]. To characterize the mRNA transcripts, sperm samples were collected in triplicate from each male and flash-frozen (fresh-frozen group) in liquid nitrogen before being stored at −80 °C. All remaining testes tissue was used to extract sperm cells for sperm quality analyses. Sperm were counted under a Zeiss Axio Imager.A2 microscope (Zeiss, Oberkochen, Germany) using a 20× objective and improved Neubauer hemocytometer. Extracted testicular sperm from each male were first diluted 100 to 200 fold, depending on initial sperm densities, in an immobilizing HBSS medium. Samples were gently inverted for ~10 s, then 10 μL was pipetted onto the hemocytometer, in duplicate per male. Sperm inside five 0.2 mm^2^ squares were counted. To obtain average concentration, diluted sperm in the five squares were summed and multiplied by 5 to estimate cells in the entire 5 × 5 grid. Sperm concentration in the 5 × 5 grid was multiplied by 10^4^ (total volume overlying the counting area) to determine the final sperm concentration based on the dilution factor.

### 4.2. Sperm Cryopreservation 

For each male, an aliquot of extracted sperm was used for evaluation of fresh sperm quality (see Section 4.3), while another aliquot was diluted to 1.0 × 10^9^ cells/mL with HBSS and 10% methanol was later added [28,29]. This sperm solution was cryopreserved in 0.5 mL cryogenic straws (~20 per male) using a Kryo 560 controlled-rate freezer (Planer Limited, Middlesex, UK) at −10 °C/min to −80 °C [29,94]. A vapor pressure liquid nitrogen storage system (MVE 815P-190F-GB, CRYO Associates, Gaithersburg, MD, USA) was used to store cryogenic samples in perpetuity. Straws were then thawed in a 40 °C water bath for 20 s. 

### 4.3. Sperm Quality Measurements

The following sperm quality traits were quantified for each male before and after cryostorage. Sperm kinematic parameters (VCL, percent motility, progressive VCL, and progressive motility) were analyzed using Computer Assisted Sperm Analysis (CASA) software (CEROS II software, Hamilton Thorne Biosciences, Beverly, MA, USA). In brief, sperm were activated in 80 µm 2X-Cel slides (Hamilton Thorne Biosciences, Beverly, MA, USA) using distilled water supplemented with 0.5% bovine serum albumin (126609-10GM, Merck Millipore, Burlington, MA, USA). Five replicate activations (for fresh and cryopreserved sperm) were conducted for each male, where video frames were analyzed at 10 s post-activation. All activations were conducted at room temperature (~20 °C).

Cell oxidative stress was assessed by flow cytometry following proprietary protocols in the Muse^®^ Oxidative Stress Kit (Luminex, Austin, TX, USA). In brief, sperm cells were diluted with 1× Assay Buffer to concentrations ranging from 1 × 10^6^ to 1 × 10^7^ cells/mL. This sperm suspension (10 uL) was then mixed with 190 uL of Oxidative Stress working solution and incubated at 37 °C for 30 min. Two cell populations were distinguished: Negative cells (ROS−) or live cells and cells exhibiting ROS activity (ROS+). A positive control (completely oxidized cells) was used to determine the division between the ROS− and ROS+ cell populations. This involved incubating sperm cells at 37 °C for 60 min in the presence of 2-methyl-1,4-naphthoquinone, 98% at 2M.

To assess sperm DNA damage, the Halomax^®^ kit (Halotech DNA, Madrid, Spain) was used. In brief, gelled aliquots of low melting point 1% agarose were placed in a 100 °C water bath until melted. Aliquots were equilibrated to 30 °C for 5 min using a water bath. Sperm were diluted to a concentration of 2.9 × 10^8^ cells/mL with HBSS before being placed on pretreated Halomax slides and covered with 22 mm × 22 mm coverslips. Slides were then placed on a precooled 4 °C metal rack to allow agarose to set for 5 min. Coverslips were slid off to the side, and the remaining steps were performed at 22 °C. Slides were then treated for 2.5 min with lysing solution before being washed in distilled water for 5 min. Thereafter, the slides were dehydrated in ethanol baths of 70% and 100% for 2 min each before being slowly air-dried and covered in foil. Samples were stained using SYBR-Green (L7011, Millipore Sigma, Burlington, MA, USA). DNA damage was assessed using a Zeiss Imager.A2 microscope (Zeiss, Oberkochen, Germany) equipped with a 40× Zeiss objective, FITC band pass filter, and X-Cite illumination system (120 LED mini+, Excelitas Technologies, Waltham, MA, USA). The presence or absence of large halos around the sperm head was assessed by taking images with ZenPro v.6.1 software (Zeiss, Oberkochen, Germany). A minimum of 500 sperm cells were assessed per male, according to Gosalvez et al. [95].

### 4.4. Total RNA Extraction, RNA-seq Library Construction, Quality Control, and Sequencing

Three biological replicates of fresh-frozen sperm samples and cryopreserved sperm samples were used for RNA extraction, which underwent the same thawing procedure (40 °C water bath for 20 s). To achieve high reproducibility, the sperm samples were homogenized using a PowerLyzer24 instrument (Qiagen, Redwood City, CA, USA). Total RNA was extracted using AllPrep DNA/RNA Kit (Qiagen, Redwood City, CA, USA) following the manufacturer’s instructions. RNA concentrations were measured with the NanoDrop OneC Microvolume Spectrophotometer (Thermo Scientific, Waltham, MA, USA). Evaluation of RNA quality was conducted using the LabChip GX Touch HT (PerkinElmer, Hopkinton, MA, USA). RNA-seq library construction was performed following the procedure described in our previous publication [96] with 500 ng of total RNA input. The size distributions of cDNA libraries were checked using the TapeStation 4200 D1000 ScreenTape (Agilent Technologies, Santa Clara, CA, USA), and library concentrations were determined by a Qubit 3.0 Fluorometer (Thermo Fisher Scientific, Waltham, MA, USA). The libraries were commercially sequenced on the Illumina NovaSeq 6000 platform with a 2 × 150 Paired-End configuration at Novogene (Novogene Corporation Inc., Sacramento, CA, USA).

### 4.5. RNA-seq QC, Genome Alignments, and Differential Gene Expression Analysis

The quality of raw RNA sequencing reads was checked by FastQC (version 0.11.6) [97]. Adapter sequences and low-quality bases were trimmed using Trimmomatic (version 0.39) [98]. RNA-seq reads shorter than 36 bp in length were excluded from subsequent analysis. The remaining high-quality reads were mapped to the blue catfish (*Ictalurus furcatus*) reference genome [99] using TopHat (version 2.1.1) [100]. To quantify gene expression levels, read counts were summarized for each gene using HTseq (version1.0) [101]. Differentially expressed genes (DEGs) between fresh-frozen sperm samples and cryopreserved sperm samples were identified using the edgeR package in R (version 3.6.4) [102]. The individual gene expression levels were quantified by Read Per Kilobase of transcript, per Million mapped reads (RPKM). Benjamini–Hochberg method [103] was applied to determine the FDR (False Discovery Rate). DEGs were detected with a cut-off of |log2(fold change)| > 2 and an adjusted *p*-value (FDR) < 0.05.

### 4.6. Functional Enrichment and Pathway Analysis of Differentially Expressed Genes in Blue Catfish Sperm before and after Cryopreservation

For the DEGs between fresh-frozen and cryopreserved sperm, the majority of them can be annotated from the closely related species channel catfish (*Ictalurus punctatus*) [104] based on homology, and the rest DEGs were annotated using eggNog-mapper [105] and Pannzer2 [106]. Metascape [107] was used to conduct Gene Ontology (GO) terms and KEGG (Kyoto Encyclopedia of Genes and Genomes) [108] pathways enrichment analysis. The gene symbol was used as input, and the zebrafish was selected as the closest model organism for analysis with the following parameters: minimum overlap of 3, *p*-value cut-off of 0.01, and minimum enrichment of 1.5. The GO terms were categorized according to biological processes, cellular components, and molecular functions. 

### 4.7. Quantitative Reverse Transcription PCR Validation of Differentially Expressed Genes

To confirm the differential gene expression identified from RNA-seq data, six candidate genes were selected to be performed with quantitative reverse transcription PCR, including *cfap206*, *rnf8*, *naa38*, *saxo2*, *tdo2b*, and *tlr5*. The *gapdh* gene was used as a reference. The reverse transcription was conducted using LunaScript^®^ RT SuperMix Kit (New England BioLabs, Ipswich, MA, USA) with 400 ng input of total RNA, following the manufacturer’s procedure. The Oligo 7.0 software (Molecular Biology Insights Inc., Cascade, CO, USA) was used to design qPCR primers for these genes (Table S3). The primers were synthesized by Eurofins (Eurofins Genomics LLC., Louisville, KY, USA), and the target size of PCR products and amplification efficiency were evaluated by 1.5% agarose gel electrophoresis. The Bio-Rad C1000 Touch Thermal Cycler with CFX96 Real-Time PCR Detection Systems (Bio-Rad Laboratories, Hercules, CA, USA) was employed to perform qRT-PCR experiments. The PCR reaction was conducted in a 20 µL mixture consisting of 8 µL of nuclease-free water, 0.5 µL of each primer (10 µmol/L), 1 µL of cDNA template, and 10 µL of Luna Universal qPCR Master Mix, using Luna^®^ Universal qPCR Master Mix (New England BioLabs, Ipswich, MA, USA). All qRT-PCR assays were conducted by incubation in 96-well plates, followed by 40 cycles at 95 °C for 15 s and 60 °C for 30 s with two technical replicates.

### 4.8. Statistical Analysis

Sperm quality measurements and qRT-PCR results were analyzed using RStudio software (version 3.6.3, Boston, MA, USA). Paired *t*-tests were performed to detect differences between the fresh-frozen and cryopreserved sperm groups. The significance threshold is *p*-value < 0.05. Reported values are the means ± standard error.

## Data Availability

The raw RNA-seq data are available at NCBI GEO (Gene Expression Omnibus) databases under the accession number GSE204687.

## Supplementary Materials

The following supporting information can be downloaded at: https://www.mdpi.com/article/10.3390/ijms23147618/s1.
