# Supplementary material for: Transcriptome Analysis Reveals Key Gene Expression Changes in Blue Catfish Sperm in Response to Cryopreservation
Source: Int J Mol Sci. 2022 Jul 10;23(14):7618. doi: 10.3390/ijms23147618 (PMC9316979; doi:10.3390/ijms23147618)
Supplement: Supplementary file 1 [file ijms-23-07618-s001.zip › FigureS1_Table_S1-S3_20220625.pdf]

Figure S1. Heatmap of differentially expressed genes (DEGs) in sperm transcriptome after cryopreservation.

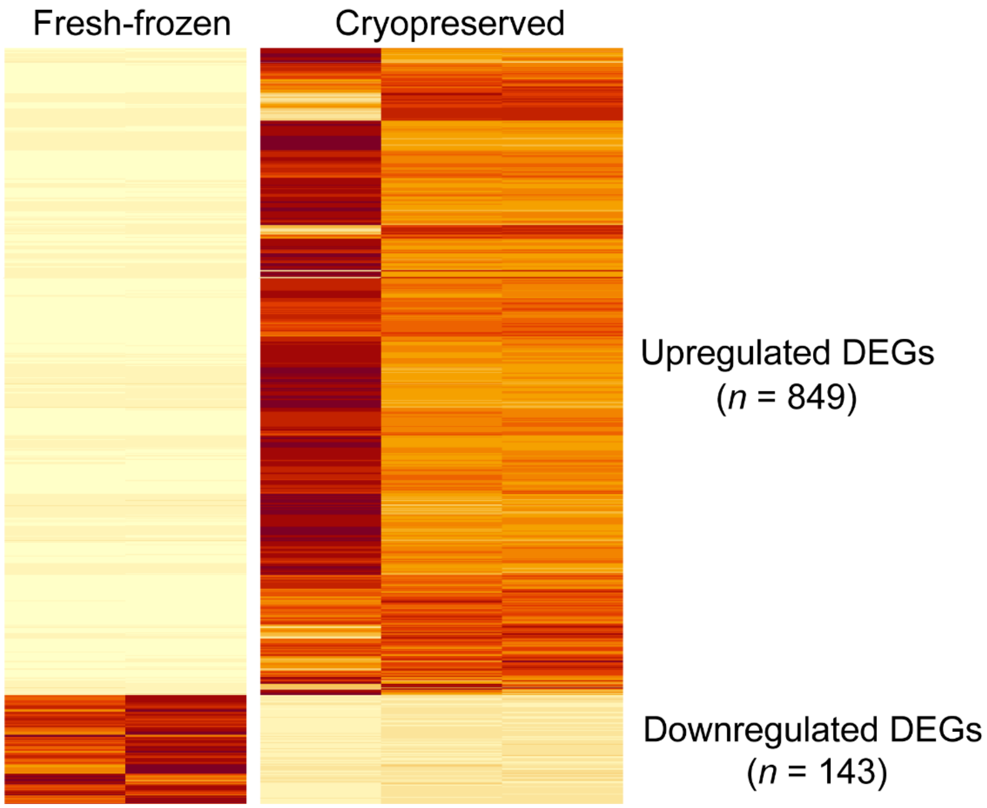

Table S1. Quality measurements of blue catfish sperm before and after cryopreservation.

Metric	Fresh sperm	Cryopreserved	*p*-value
Motile VCL (μm/s)	172.48 ± 10.16	133.83 ± 14.89	0.0090
Percentage of motility (%)	56.67 ± 11.84	41.30 ± 14.46	0.0758
Progressive curvilinear velocity (μm/s)	183.5 ± 8.55	150.18 ± 14.37	0.0090
Percentage of Progressive VCL (%)	52.47 ± 11.98	34.76 ± 12.53	0.0472
Oxidative level (ROS+ %)	14.39 ± 2.31	34.03 ± 3.56	0.0495
DNA fragmentation (%)	11.33 ± 2.51	42.67 ± 7.97	0.0495

Significance level was detected by Kruskal-Wallis test and cutoff is $p < 0.05$. The values are shown with Mean ± SD.

Table S2. Sperm RNA sequencing yield, quality filtering, and alignment statistics.

Sample ID	Number of raw reads	Number of filtered reads	Mapping percentages to blue catfish genome
C2_Cryo_sperm	34,561,760	33,714,312	78.1%
C3_Cryo_sperm	19,326,022	18,455,548	81.5%
C5_Cryo_sperm	29,746,210	29,002,382	78.8%
F2_Fresh_sperm	54,007,688	49,703,360	73.7%
F3_Fresh_sperm	74,715,160	70,866,418	79.0%
F5_Fresh_sperm	80,395,632	78,196,414	< 20%, excluded

Table S3. Primer sequences for quantitative reverse transcription PCR validation in blue catfish sperm samples.

Gene name	5'- 3' primer sequence	PCR product size (bp)
Saxo2-F	GTACAGCCAGGAGTTTGTCCC	235
Saxo2-R	AAATCATCCTGGAATGTTGTGG	
Cfap206-F	AAGGCAGACGAGTATATCGAG	129
Cfap206-R	TGTCTCACCTGACTGCAT	
TLR5-F	AGAACCTACATCCAGACCAG	116
TLR5-R	ACTTGCTTGATTTTGGTGT	
NAA38-F	TCGGAGCAGTGTGTATATACGTG	187
NAA38-R	CAGCCTGAGAGAACGAGT	
Rnf8-F	ACGTGGACTGAATGTCACC	138
Rnff8-R	ACCCACACTCCATTTAGGC	
Tdo2b-F	ATTTCAAAACCAATCCCAG	122
Tdo2b-R	AGCATTAATAACCTTATCCAG	
Hectd1-F	TATGCTGCCTGTTTTGGACGA	125
Hectd1-R	TCATTATGTCCCCGCTCCC	
GAPDH-F	AGACTTCAATGGAGATACTCA	111
GAPDH-R	CTGTAGCCAAACTCGTTG	
